# Observations on “Trench Nephritis” at a British General Hospital in France

**Published:** 1918-03

**Authors:** 


### MEDICAL

**_Observations on "Trench Nephritis" at a British General Hospital in France_** (A Preliminary note of an article to appear elsewhere). By Captain R. FITZ, M. O. R. C., U. S. A., from the Medical Division of U. S. Army Base Hospital No 5 (Major R. I. LEE, M. O. R. C., U. S. A., Medical Director.)

"Trench Nephritis" has been defined by General Sir John Rose Bradford as a type of nephritis occurring amongst soldiers characterized by the frequent presence of bronchitis and dyspnea, the suddenness of onset of uremic manifestations, the rapid subsidence of well marked renal dropsy, the rarity of occurrence of inflammatory complications, and by an extraordinarily low mortality.

A statistical study of the medical cases admitted to United States Base Hospital No. 5 during June, July, August, and September, 1917 was undertaken with a view to determining the frequency of this type of nephritis in a typical general war hospital during the summer months. It represented about 5 per cent of the medical diseases admitted, and was strikingly more frequent than acute nephritis in a general civilian hospital.

The cases of "Trench Nephritis" admitted were analyzed with a view to demonstrating the age incidence of the disease amongst the medically sick, its relation to the patient's length of service in France, and to determining whether any epidemic foci of the disease could be discovered. In confirmation of other similar statistical studies, "Trench Nephritis" in this hospital occurred most commonly amongst relatively aged soldiers who had been in France for short periods of time. It occurred to a less extent amongst the youngest troops and amongst the most seasoned. It appeared to be pandemic and was not limited to any single group or groups of soldiers.

Studies on renal function were made in 18 typical cases of "Trench Nephritis" with a view to inquiring further into the cause of the characteristic symptoms and course of the disease. The dyspnea was not due to acidosis, though a slight acidosis was common. The edema was intimately related to chloride retention and was independent of high blood pressure, albuminuria, or urea retention. The epileptiform convulsions observed, occurred in relatively mild cases without markedly abnormal kidney function and were not associated with urea retention, oliguria, or anuria. The cases which were complicated by convulsions usually had hypertension of varying degree, and all showed more or less chloride retention. It seems possible that they were not necessarily " uremic " in nature, but depended upon some circulatory disturbance or cerebral edema.

Fever at onset was observed in seven cases and suggested that the disease in certain instances might be of infectious origin, an impression which was confirmed by the tendency to relapse which certain cases showed. Two types of relapse were encountered, the one febrile in character and associated with symptoms of " Trench Fever ", the other having in addition to fever, a concomitant rise in blood pressure, hematuria and return of such symptoms of intoxication as headache and nausea.

The immediate prognosis of " Trench Nephritis " was most accurately obtained by determining the accumulation of urea in the blood. This was greatest in the severest cases, tended to diminish at times when the albuminuria and hypertension diminished, and was independent of edema and chloride retention. It would be reasonable to suppose that fatal cases with or without suppression of urine would show extremely high blood urea concentration.

The ultimate prognosis of the disease is less certain. Of the sixteen cases in which data were obtained, seven were fit for active military duty either at home or abroad within four months after the onset of symptoms, two had been discharged from the army on account of physical unfitness, at intervals up to eight months after the onset of symptoms, and seven cases were still in hospital. In no case was there evidence for believing that the disease showed any chronic progressive tendency, and in every case it seemed probable that recovery would eventually occur. It was impossible to predict from the severity of signs or symptoms at onset how long the disease would take to heal.

Since the disease has a low mortality, treatment is relatively unimportant. However, since edema is associated with chloride retention, and the severest cases with urea retention, it is reasonable to give patients with " Trench Nephritis " a diet low in chloride and nitrogen. When the blood urea has become normal, and the edema has disappeared, the cases should be treated as cases of chronic nephritis and observed carefully until they are well.

